# Association of tobacco purchasing behaviors with tobacco use by user groups during the COVID-19 pandemic: A mixed methods analysis

**DOI:** 10.1371/journal.pone.0313566

**Published:** 2024-11-21

**Authors:** Nikita G. Kute, David L. Ashley, Claire A. Spears, Amy L. Nyman, Katherine C. Henderson, Vuong V. Do, Jidong Huang, Lucy Popova

**Affiliations:** 1 New York State Department of Health, Health Research Inc., Albany, New York, United States of America; 2 Department of Population Health Sciences, School of Public Health, Georgia State University, Atlanta, Georgia, United States of America; 3 Department of Health Policy & Behavioral Sciences, School of Public Health, Georgia State University, Atlanta, Georgia, United States of America; 4 Center for Tobacco Control Research and Education, University of California San Francisco, San Francisco, California, United States of America; Mattu University, ETHIOPIA

## Abstract

**Purpose:**

To understand changes in purchasing behaviors and use of tobacco products such as e-cigarettes and cigarettes among different tobacco user groups during the COVID-19 pandemic using a mixed methods approach.

**Methods:**

A quantitative online survey was conducted in October-November 2020 using a national probability sample of US adults (N = 1,460) comprising exclusive cigarette smokers (n = 1,080), dual users of both cigarettes and e-cigarettes (n = 143), and exclusive e-cigarette users (n = 237). Simultaneously, ten online focus groups were conducted with 61 adults in the Atlanta, GA area including exclusive smokers (n = 16), current E-cigarette users (n = 22), and transitioning (recently quit or currently quitting) smokers and/or E-cigarette users (n = 23).

**Results:**

From the survey, dual users vs. exclusive smokers had higher odds of buying cheaper cigarette brands (adjusted odds ratio (aOR) 2.50, 95% confidence interval (CI) = 1.49, 4.20), buying cigarettes online (aOR = 2.79; 95% CI = 1.02, 7.69), buying from Indian Reservations (aOR = 3.99; 95% CI = 2.07, 7.69), buying fewer cigarettes than normal (aOR = 4.01; 95% CI = 2.42, 6.65) and buying other tobacco products (aOR = 4.44; 95% CI = 2.24, 8.79). From the focus groups, participants perceived reduced accessibility, fear of contracting COVID-19, rising prices, and convenience to influence their purchasing behaviors and tobacco use.

**Conclusions:**

Exclusive and dual users differed in their tobacco purchasing behaviors during the COVID-19 pandemic, such that dual users were more likely to change their purchasing behaviors (e.g., buying other tobacco products) than exclusive users. Educational campaigns and public health workers may promote interventions targeting dual users either to switch to reduced-risk products or quit smoking, particularly during stressful societal situations such as the COVID-19 pandemic.

**Implications:**

The findings inform public health educators and policymakers to develop policies and interventions carefully tailored for tobacco user groups targeting the perceived factors influencing purchasing behaviors during challenging situations affecting tobacco product availability.

## Introduction

The COVID-19 outbreak, first detected in December 2019, rapidly spread around the globe and was declared a pandemic by the World Health Organization in March 2020 [1]. The year 2020 challenged societal norms with surging cases and deaths, implementation of preventive measures such as lockdowns, and worldwide efforts to develop treatments and vaccines against the virus [1]. All these factors added to the economic hardship which further escalated mental and physical stress during 2020 [2,3].

Tobacco use in the form of smoking or vaping is commonly associated with lung diseases in addition to other diseases such as heart disease or diabetes [4,5]. COVID-19, a respiratory illness, was also found to be associated with increased severe complications and death among smokers [6]. The combination of tobacco use and contracting COVID-19 increased public health concerns during the pandemic and points to a need to evaluate both health threats combined instead of merely as independent issues.

Preventive measures implemented across almost all countries in the world to curb the spread of COVID-19, such as lockdowns, use of face masks, social distancing, and quarantine orders [7], led to abrupt changes in lifestyle and severely impacted the economic systems and consumer markets in many countries [8]. Perceptions of the risk of COVID-19 infection, rising anxiety and fear, and uncertainties regarding the availability of necessary commodities led to changes in consumer behaviors such as increased purchasing quantity or reduced store visits [8–10]. Considering the highly addictive nature of tobacco products [11] and the association of tobacco use with stress and anxiety during the pandemic [12,13], studying tobacco product purchasing behaviors among tobacco users during the COVID-19 pandemic can provide insight into product appeal and user perception under periods of societal stress.

Most studies of purchasing behaviors for tobacco products during the COVID-19 pandemic have focused on behaviors such as buying online, changing quantity, or purchasing alternate tobacco products among cigarette users and/or e-cigarette users [14–17]. However, other purchasing behaviors, such as buying more packs per store visit, buying cheaper cigarette brands, buying from alternate sources such as Indian reservations, going to stores more often, and changing the brand, type, flavor, or place of purchase of tobacco products remain unexplored. In addition, studies have found that the pandemic changed patterns of tobacco use differently for different tobacco user groups (e.g., exclusive smokers, exclusive e-cigarette users, or dual users of e-cigarettes and cigarettes). For example, exclusive smokers reported that they increased their smoking, e-cigarette users reported to have reduced their vaping, and dual users reported to have increased smoking instead of vaping during the pandemic [12,18,19]. However, little is known about how these groups changed their purchasing behavior. Previous studies identified purchasing behaviors among cigarette or e-cigarette users [14–17]. However, since smokers and e-cigarette users can have exclusive or dual use patterns, limited research exists in understanding whether there are significant differences in tobacco purchasing behaviors across these tobacco user groups and if changes in purchasing behaviors predict their tobacco product use during the pandemic [20].

Decision making when purchasing tobacco products is a complex behavior. A mixed methods approach combining findings from both quantitative and qualitative studies can reveal broader perspectives behind such complex behaviors than qualitative or quantitative findings alone [21]. In this mixed methods study, we examined various purchasing behaviors for e-cigarettes and cigarettes and evaluated their differences among tobacco user groups such as exclusive smokers, exclusive e-cigarette users, and dual users of cigarettes and e-cigarettes. We also analyzed whether tobacco purchasing behaviors were associated with tobacco product use among adult smokers and e-cigarette users in the US. We conducted a quantitative national probability survey with adult cigarette and/or e-cigarette users. In parallel, we conducted focus groups with a different purposive sample of smokers and e-cigarette users, which identified important themes related to purchasing behaviors and tobacco use during the peak phase of the COVID-19 pandemic from the users’ point of view. The themes that emerged in the focus groups highlighted the possible influencing factors behind the findings of the quantitative analysis.

## Methods

### Quantitative study: Survey

#### Study population

A survey was conducted in October-November 2020 using a non-institutionalized nationally representative random sample of US adults from the Ipsos Public Affairs KnowledgePanel. Detailed information on the panel can be found elsewhere [22]. The survey examined the perceived effects of the COVID-19 pandemic on the availability of tobacco products, purchasing behavior, and use of tobacco products as well as perceptions and knowledge of smokers and e-cigarette users. The target population for the survey included adults aged 18+ years who were current cigarette smokers or e-cigarette users or recent quitters (since February 2020, marking the beginning of the COVID-19 pandemic) of these products. The recruitment period for the study included a soft launch period from 10/23/2020 to 10/25/2020 and a main recruitment period from 10/29/2020 to 11/9/2020. Consent was waived off for the survey as participants were recruited from the KnowledgePanel. The study was deemed exempt from federal human subject’s research regulations by the Georgia State University Institutional Review Board. The final sample for this study included 1,460 participants categorized as exclusive smokers (smoked 100 cigarettes in their lifetime, now smoke every day or some days, and do not currently use e-cigarettes; n = 1080), dual users of cigarettes and e-cigarettes (smoked 100 cigarettes in their lifetime, currently smoke every day or some days, and currently use e-cigarettes every day or some days, n = 143), and exclusive e-cigarette users (currently use e-cigarette every day or some days and currently do not smoke, n = 237). For some analyses, we also classified people into current e-cigarette users (exclusive e-cigarette users and dual users) and current smokers (exclusive smokers and dual users). Study-specific post-stratification weights were computed to allow the data to be proportional to the National Health Interview Survey data.

#### Measures

*E-cigarette Purchasing Behavior*. The survey included 8 categorical items related to e-cigarette purchasing behavior; four of these were asked using three category response options (*Increased*, *Stayed the same*, *or Decreased*) starting with “*Since February*, *2020*, *please specify whether each of the following has increased*, *stayed the same*, *or decreased*: *1) the amount of e-liquid or pods you buy at once; 2) your typical nicotine concentration; 3) your typical package size (for example number of pods per package*, *size of e-liquid container*, *etc*.*)* and *4) how often you typically purchase your product*.*”* The other four items were asked using binary (*yes/no*) response options, starting with “*Since February 2020*, *have you changed*: *1)the typical brand of the electronic vapor product that you use; 2) the type of electronic vapor product that you use; 3) the typical flavor of e-liquid that you use;* and *4) where you typically purchase your electronic vapor product*.*”*

*Cigarette Purchasing Behavior*. The survey included 8 binary items related to cigarette purchasing behavior, all of which were asked with binary (*yes/no*) response options, starting with “*Since February 2020*, *have you been*: *1) buying more packs per store visit; 2) buying cartons instead of packs; 3) buying cheaper cigarette brands; 4) buying cigarettes online; 5) going to the store more frequently to buy cigarettes; 6) buying cigarettes from an Indian Reservation; 7) buying fewer cigarettes than normal;* and *8) buying other tobacco products instead of cigarettes (e*.*g*., *filtered cigars*, *cigarillos*, *smokeless tobacco)”*. Questions related to cigarette purchasing behavior were adapted from a survey developed by the University of Vermont TCORS (UTV TCORS) research team, while the questions for e-cigarette purchasing behaviors were developed by the Georgia State University research team [23].

*Change in E-cigarette Use*. Participants who reported current use of e-cigarettes were asked, “*How has your electronic vapor product use changed since February 2020 (the start of the COVID-19 pandemic)*?*”* with categorical response options “*I increased my electronic vapor product use*, *my electronic vapor product use has stayed the same* and *I decreased my electronic vapor product use”*.

*Change in Cigarette Use*. Participants who reported being current smokers were asked, “*How has your cigarette use changed since February 2020 (the start of the COVID-19 pandemic*?*”* with categorical response options “*I increased my cigarette use*, *my cigarette use has stayed the same* and *I decreased my cigarette use”*. These questions were adapted from UVT TCORS [23].

*Nicotine Addiction Score (PROMIS Scale)*. Nicotine addiction is a continuous variable measured for e-cigarette users and smokers separately. We used 4 items from the PROMIS scale for nicotine addiction, with response options ‘*Never’*, *‘Rarely’*, *‘Sometimes’*, *‘Often’*, *and ‘Always’* for each item [24,25]. Current e-cigarette users were asked, “*Please rate how often each of the following statements applies to you*, *1) I find myself reaching for electronic nicotine products without thinking about it*. *2) I drop everything to go out and buy electronic nicotine products (or e-juice)*. *3) When I haven’t been able to vape for a few hours*, *the craving gets intolerable*. *4) I vape more before going into a situation where vaping is not allowed”*. Current smokers were asked, “*Please rate how often each of the following statements applies to you*, *1) I find myself reaching for cigarettes without thinking about it*. *2) I drop everything to go out and buy cigarettes*. *3) When I haven’t been able to smoke for a few hours*, *the craving gets intolerable*. *4) I smoke more before going into a situation where smoking is not allowed”*. The sum of the raw scores for the 4 items for both cigarette and e-cigarette ranged from 4 to 20. We converted the sum of raw scores into T-scores with a mean of 50 and standard deviation of 10 (standard metric system for PROMIS nicotine addiction scale) [26] using the short form conversion table.

*Socio-Demographics*. Demographic variables included categorical variables for age group, gender, race/ethnicity, income level, and employment status. To account for different tobacco control environments on a state level, we controlled for the state participants lived in. The variables were collected from Ipsos Public Affairs’ profile surveys.

#### Data analysis

Data from the survey were analyzed using SAS version 9.4. Weighted percentages and unweighted frequencies were reported to characterize the sample. Bivariate analysis was conducted using Chi-Square test to assess the hypothesis of association between tobacco user groups and socio-demographic variables and nicotine addiction for cigarettes and e-cigarettes. Following analyses were conducted: 1) The adjusted association between tobacco user groups and e-cigarette purchasing behavior: eight binary logistic regression models were conducted, one for each e-cigarette purchasing behavior as outcome and tobacco user group as exposure controlling for socio-demographic variables, state of residence and e-cigarette nicotine addiction as confounding factors; for ease of interpretation of results and to focus the findings on ‘increase in tobacco purchasing behavior’, we converted the three category responses for the four items (amount, nicotine concentration, package size, and purchasing frequency) assessing e-cigarette purchasing behavior into two categories by combining the responses ‘stayed the same’ and ‘decreased’ into one response to form a binary variable (Increased vs. Stayed the same or decreased). 2) The adjusted association between tobacco user groups and cigarette purchasing behavior: Eight binary logistic regression models were conducted, one for each cigarette purchasing behavior as outcome and tobacco user group as exposure controlling for socio-demographic variables, state of residence and cigarette nicotine addiction as confounding factors. 3) The association between e-cigarette purchasing behaviors and change in e-cigarette use: single multinomial logistic regression model with change in e-cigarette use as outcome and e-cigarette purchasing behaviors as exposure, controlling for other e-cigarette purchasing behaviors, socio-demographic variables, states of residence and e-cigarette nicotine addiction. 4) The association between cigarette purchasing behaviors and change in smoking: single multinomial logistic regression model was conducted with change in cigarette use as outcome and cigarette purchasing behaviors as exposures controlling for other cigarette purchasing behaviors, socio-demographic variables, state of residence and cigarette nicotine addiction as confounding factors. Adjusted Odds Ratios (aOR) and 95% confidence intervals (CI) were reported. Analyses were conducted using SAS SURVEYFREQ procedures and weighted using the computed study-specific post-stratification weights to allow the data to be proportional to the National Health Interview Survey and account for the survey design, oversampling, or non-response.

### Qualitative study: Focus groups

#### Study population

Detailed methods for the focus group study are described elsewhere [12]. Briefly, we conducted 10 online focus groups (via Zoom) in October-November 2020 with different participants from those who responded to the quantitative survey. The recruitment period for the focus group study was from 10/23/2020 to 11/05/2020. Inclusion criteria were being 18 years or older and being current cigarette smokers or e-cigarette users or having quit cigarettes or e-cigarettes since February 2020. All participants who completed the screener and were chosen for inclusion were provided with a link to the online consent form and a pre-participation survey. Participants who provided informed consent electronically and indicated willingness to participate proceeded to the pre-participation survey that collected additional demographic and past tobacco use information. Those who completed the pre-participation survey were then provided with a link to the online focus group meeting. The study was approved by Georgia State University IRB. Participants were compensated with $50 for their time. We conducted four focus groups with exclusive smokers (n = 16; smoked 100 or more lifetime cigarettes and currently smoking but not currently using e-cigarettes and not currently trying to quit smoking); three with current e-cigarette users (n = 22; current e-cigarette users, may also be current smokers but not attempting to quit smoking); and three with transitioning smokers and/or e-cigarette users (n = 23; current smokers or e-cigarette users who are trying to quit smoking or had quit since February 2020). Each focus group had between 2 and 8 participants (median = 7). One group had multiple no-shows and had only 2 participants in the session. The focus groups lasted 45–82 minutes. Moderators from John Snow Inc. [27] facilitated the focus group sessions using a semi-structured interview guide developed by the GSU research team. The guide covered discussion topics focused on cigarette and e-cigarette use in the context of COVID-19. In this study, we focus on the themes identified for tobacco purchasing behaviors and the differences in purchasing behavior across tobacco user groups.

#### Data analysis

The focus group sessions were audio recorded, transcribed verbatim, and analyzed using NVivo version 12.0 software, using a thematic analysis method [28]. An initial codebook was developed by co-author LP after having reviewed the transcripts. Using the drafted codebook, co-author KH and first author NK independently coded one randomly selected transcript. The three authors then discussed together the discrepancies and revised the codebook. NK and KH then independently coded the remaining transcripts using the finalized codebook. The extracted codes were then distributed among the co-authors to review and write summary memos. The authors then discussed the memos together to identify themes related to purchasing behaviors and tobacco use during the COVID-19 pandemic and the factors influencing them. The descriptive analysis for the focus group sample was conducted using SPSS software.

## Results

### Quantitative study: Survey

#### Participant characteristics

In the quantitative survey sample, approximately half of the participants were male (54.1%), half were aged 18 to 44 years (50.6%) and two-thirds were non-Hispanic White (71.8%). Most of the participants were employed (62.9%) and about half had an annual household income of $30,000-$99,999 (47.3%) (Table 1). Nicotine addiction scores for E-cigarette users had a mean of 42.2 and a standard deviation of 9.6, while those for smokers had a mean of 46.3 and a standard deviation of 9.5. Except for sex and e-cigarette nicotine addiction, all the socio-demographic variables and cigarette nicotine addiction scores were significantly associated with tobacco user groups in bivariate associations (Table 1).

**Table 1:** Participant characteristics by tobacco user groups (survey study).

Participant characteristic	Exclusive E-cigarettes Users N = 237, n(%)	Dual Users N = 143, n(%)	Exclusive Smokers N = 1080, n(%)	Total Sample N = 1460, n(%)	Rao Scott Chi-Sq P value
**Sex**					
** Male**	134 (57.2)	81 (55.8)	597 (52.9)	812 (54.1)	.6244
** Female**	103 (42.9)	62 (44.2)	483 (47.1)	648 (45.9)	
**Age Group**					
** 18–29 years**	43 (39.3)	22 (33.2)	51 (13.6)	116 (20.9)	**< .0001**
** 30–44 years**	69 (26.3)	51 (39.8)	242 (28.8)	362 (29.7)	
** 45–59 years**	63 (23.3)	39 (17.5)	351 (31.8)	453 (28.4)	
** 60+ years**	62 (11.2)	31 (9.5)	436 (25.7)	529 (20.9)	
**Race**					
** White, NH**	195 (81.7)	101 (61.9)	819 (70.8)	1115 (71.8)	**.0098**
** Black, NH**	12 (4.4)	14 (10.1)	124 (13.9)	150 (11.6)	
** Other, NH**	14 (7.2)	18 (17.6)	82 (9.5)	114 (10.0)	
** Hispanic**	16 (6.7)	10 (10.4)	55 (5.9)	81 (6.6)	
**Employment**					
** Working**	160 (71.8)	97 (68.2)	605 (59.5)	862 (62.9)	**.0114**
Non-Working 1*	14 (9.1)	10 (12.9)	63 (8.6)	87 (9.2)	
Non-Working 2*	63 (19.1)	36 (18.9)	412 (31.9)	511 (27.9)	
**Household Income**					
** <$30K**	48 (17.7)	41 (27.0)	333 (30.2)	422 (27.4)	**.0054**
** $30K-$99,999**	123 (48.1)	70 (44.1)	549 (47.7)	742 (47.3)	
** ≥$100K**	66 (34.1)	32 (28.9)	198 (22.1)	296 (25.3)	
**E-cigarette users Nicotine Addiction Score** **N = 380**					
**Mean (SD)**	43 (9.4)	40.9 (9.9)		42.2 (9.6)	.4616^**#**^
**Cigarette Users Nicotine Addiction Score** **N = 1223**					
**Mean (SD)**		47.6 (10.3)	46.1 (9.4)	46.3 (9.5)	**.0317** ^**#**^

n–Unweighted Frequency; %—Weighted Row Percentages.

NH- Non-Hispanic.

NA- Not applicable.

SD–Standard Deviation.

Nicotine addiction scores are the sum of raw scores for nicotine addiction scale items converted into T scores with a mean of 50 and a standard deviation of 10.

† Rao Scott Chi Square P values. Bolded P values are statistically significant at the significance level less than or equal to 0.05.

# P-values were calculated using bivariate multinomial logistic regressions.

*Non-Working 1 = Laid off / Looking for work.

*Non-Working 2 = Retired / Disabled / Other.

#### Association between purchasing behaviors and tobacco user groups

*E-cigarette Purchasing Behavior*. Most e-cigarette users reported no change in their e-cigarette purchasing behavior during the COVID-19 pandemic. For those who changed their behavior, no significant associations were found between tobacco user groups and e-cigarette purchasing behaviors during COVID-19, after controlling for socio-demographics, state of residence, and nicotine addiction (Table 2).

**Table 2:** E-cigarette purchasing behavior among exclusive e-cigarette users and dual users of e-cigarette and cigarettes (survey study).

E-cigarette purchasing behavior	Status	Exclusive E-cigarette User N = 237, n(%)	Dual User N = 143, n(%)	Total Sample N = 380, n(%)	Dual Users vs Exclusive E-Cigarette Users aOR^¶^ (CI)
**Amount of e-liquid or pods^#^**	Increased	29 (14.4)	12 (10.1)	41 (12.7)	0.65 (0.28, 1.53)
	Stayed the same	190 (77.5)	110 (78.9)	300 (78.1)	Ref
	Decreased	18 (8.1)	21 (10.9)	39 (9.2)	
**Nicotine Concentration^#^**	Increased	3 (1.4)	6 (5.4)	9 (2.9)	4.74 (0.65, 34.65)
	Stayed the same	196 (84.9)	120 (86.0)	316 (85.4)	Ref
	Decreased	38 (13.6)	17 (8.6)	55 (11.7)	
**Package Size^#^**	Increased	16 (7.2)	7 (4.2)	23 (6.0)	0.49 (0.15, 1.57)
	Stayed the same	206 (87.8)	116 (84.9)	322 (86.7)	Ref
	Decreased	15 (5.0)	20 (10.9)	35 (7.3)	
**Purchasing Frequency^#^**	Increased	20 (9.1)	12 (11.7)	32 (10.1)	0.91 (0.33, 2.51)
	Stayed the same	178 (71.8)	105 (75.9)	283 (73.4)	Ref
	Decreased	39 (19.2)	26 (12.4)	65 (16.5)	
**Brand^+^**	Yes	23 (13.8)	21 (17.3)	44 (15.2)	1.39 (0.58, 3.35)
**Type^+^**	Yes	19 (11.6)	20 (17.8)	39 (13.9)	1.72 (0.69, 4.31)
**Flavor^+^**	Yes	25 (13.1)	26 (21.6)	51 (16.4)	1.82 (0.79, 4.19)
**Place of purchase^+^**	Yes	42 (20.6)	29 (21.7)	71 (21.0)	0.94 (0.45, 1.97)

n = Unweighted Frequency; % = Weighted Row Percentages.

^#^ Reported percentages are for Increased vs. Stayed the same or decreased. The three categories for these variables were converted into two categories by condensing ‘Stayed the same’ and ‘Decreased’ responses (Increased vs. Decreased or Stayed the same).

^+^ Reported percentages are for Yes vs. No.

† Rao Scott Chi Square P values. Bolded P values are statistically significant at the significance level of 0.05.

aOR–Adjusted Odds Ratio for Dual Users vs. Exclusive E-cigarette users; CI–Confidence Intervals.

¶ Adjusted for E-cigarette user’s nicotine addiction, age, gender, race, household income, employment status, and state of residence.

*Cigarette Purchasing Behavior*. Like for e-cigarette purchasing behaviors, overall, most smokers reported no change in their cigarette purchasing behavior during the COVID-19 pandemic (Table 3). For those who changed their behavior, there were significantly higher odds for dual users vs. exclusive smokers to buy cheaper cigarette brands (aOR = 2.50; 95% Confidence Intervals (CI) = 1.49, 4.20), buy online (aOR = 2.79; 95% CI = 1.02, 7.69), buy from an Indian Reservation (aOR = 3.99; 95% CI = 2.07, 7.69), buy fewer cigarettes than normal (aOR = 4.01; 95% CI = 2.42, 6.65), and buy alternative tobacco products (aOR = 4.44; 95% CI = 2.24, 8.79) during the COVID-19 pandemic, after adjusting for nicotine addiction, state of residence and socio-demographic variables (Table 3).

**Table 3:** Cigarette purchasing behavior by tobacco user groups (survey study).

Cigarette purchasing behavior	Exclusive Smoker N = 1080, n (%)	Dual User N = 143, n (%)	Total Sample N = 1223, n (%)	Dual users vs Exclusive Smokers aOR^¶^ (CI)
**Buying more packs per store visit**	225 (20.3)	40 (30.0)	265 (21.8)	1.39 (0.81, 2.36)
**Buying cartons instead of packs^a^**	297 (24.4)	28 (25.6)	325 (24.6)	1.04 (0.59, 1.84)
**Buying cheaper cigarette brands**	172 (15.1)	37 (30.9)	209 (17.5)	**2.50 (1.49, 4.20)**
**Buying online^a^**	21 (1.9)	10 (7.8)	31 (2.8)	**2.79 (1.02, 7.69)**
**Increased store visits to buy cigarettes**	136 (14.5)	33 (26.4)	169 (16.3)	1.43 (0.79, 2.59)
**Buying from Indian Reservation^a^**	65 (4.6)	19 (15.4)	84 (6.3)	**3.99 (2.07, 7.69)**
**Buying fewer cigarettes than normal^a^**	179 (14.9)	51 (38.8)	230 (18.6)	**4.01 (2.42, 6.65)**
**Buying other tobacco products^a^**	45 (4.5)	24 (21.9)	69 (7.2)	**4.44 (2.24, 8.79)**

n = Unweighted Frequency; % = Weighted Row Percentages.

Reported frequency percentages are for Yes vs. No.

aOR–Adjusted Odds Ratio for Dual Users vs. Exclusive Smokers; CI–Confidence Intervals.

¶ Adjusted for smokers’ nicotine addiction, age, gender, race, household income employment status, and state of residence.

a: One missing in the total sample size. N = 1222 (One missing).

Bolded odds ratios are significant at alpha less than or equal to 0.05.

#### Association between e-cigarette purchasing behavior and e-cigarette use during the COVID-19 pandemic

After controlling for other e-cigarette purchasing behaviors, nicotine addiction, tobacco user groups, state of residence, and socio-demographic factors, the odds of decreased e-cigarette use vs. no change were higher for those who increased their purchasing package size (aOR = 6.90; 95% CI = 1.33, 35.84). The odds of increased E-cigarette use vs. no change were higher for those who increased their purchasing frequency (aOR = 8.21; 95% CI = 2.53, 26.62) and for those who changed their brand of E-cigarette product (aOR = 5.55; 95% CI = 1.07, 28.86) (Table 4).

**Table 4:** Adjusted association between change in e-cigarette use and e-cigarette purchasing behaviors during the COVID-19 pandemic among e-cigarette users including exclusive and dual users (survey study).

E-cigarette Purchasing Behavior	Status	Change in E-cigarette use			aOR^¶^ (95% CI)	
		Increased N = 60 n(%)	No change N = 271 n(%)	Decreased N = 44 n(%)	Increased vs No change	Decreased vs No change
**Amount of e-liquid or pods^#^**	Increased	13 (21.7)	23 (9.8)	4 (16.1)	1.01 (0.27, 3.83)	2.43 (0.47, 12.51)
	Stayed the same	45 (74.3)	235 (84.9)	17 (42.4)	Ref	Ref
	Decreased	2 (3.9)	13 (5.3)	23 (41.5)		
**Nicotine Concentration^#^**	Increased	3 (8.9)	4 (1.3)	2 (4.6)	0.81 (0.14, 4.69)	3.87 (0.34, 44.36)
	Stayed the same	47 (79.1)	235 (88.5)	30 (76.9)	Ref	Ref
	Decreased	10 (11.9)	32 (10.2)	12 (18.4)		
**Package Size^#^**	Increased	10 (13.8)	8 (3.1)	4 (12.9)	1.20 (0.27, 5.44)	**6.90 (1.33, 35.84)**
	Stayed the same	48 (84.8)	250 (92.6)	22 (54.3)	Ref	Ref
	Decreased	2 (1.4)	13 (4.3)	18 (32.9)		
**Purchasing Frequency^#^**	Increased	18 (33.7)	9 (3.7)	4 (13.0)	**8.21 (2.53, 26.62)**	3.26 (0.65, 16.27)
	Stayed the same	39 (64.1)	230 (82.3)	11 (29.4)	Ref	Ref
	Decreased	3 (2.2)	32 (13.9)	29 (57.6)		
**Changed Brand^+^**	Yes	17 (38.1)	22 (10.9)	3 (4.9)	**5.55 (1.07, 28.86)**	0.29 (0.03, 2.97)
**Changed Type^+^**	Yes	13 (32.1)	22 (10.8)	3 (4.9)	0.32 (0.05, 1.98)	0.22 (0.02, 2.17)
**Changed Flavor^+^**	Yes	17 (38.4)	29 (11.9)	5 (11.8)	1.62 (0.47, 5.58)	3.04 (0.45, 20.47)
**Changed Place of purchase^+^**	Yes	17 (38.3)	47 (18.1)	5 (10.0)	0.86 (0.23, 3.24)	0.32 (0.06, 1.72)

n = Unweighted Frequency; % = Weighted Row Percentages.

aOR: Adjusted Odds Ratio.

CI: 95% Confidence Interval.

¶ Adjusted for all other E-cigarette purchasing behaviors, E-cigarette nicotine addiction, tobacco user groups, age, gender, race, household income, state of residence, and employment status.

# Increased vs. Decreased or No change.

+ Yes vs. No.

Bolded odds ratios are significant at the alpha less than or equal to 0.05.

#### Association between cigarette purchasing behavior and smoking during the COVID-19 pandemic

After controlling for other cigarette purchasing behaviors, nicotine addiction, tobacco user group, state of residence, and socio-demographic factors, the odds of increased smoking vs. no change were higher for those who bought more packs per store visit (aOR = 2.54; 95% CI = 1.57, 4.10) and those who were going to the store more frequently to buy cigarettes (aOR = 5.19; 95% CI = 2.95, 9.11). The odds of decreased smoking vs. no change were higher for those who bought cigarettes online (aOR = 4.12; 95% CI = 1.07, 15.82), and for those who bought fewer cigarettes than normal (aOR = 29.35; 95% CI = 17.45, 49.36) during the COVID-19 pandemic (Table 5).

**Table 5:** Adjusted associations between change in cigarette use and cigarette purchasing behaviors during the COVID-19 pandemic among current smokers (survey study).

Cigarette Purchasing Behavior	Change in smoking			aOR^¶^ (95% CI)	
	Increased N = 189 n (%)	No change N = 839 n(%)	Decreased N = 184 n(%)	Increased vs No change	Decreased vs No change
**More packs per store visit**	92 (44.5)	144 (18.0)	27 (14.5)	**2.54 (1.57, 4.10)**	1.05 (0.50, 2.20
**Cartons instead of packs**	66 (31.6)	222 (24.8)	32 (14.5)	1.14 (0.66, 1.96)	0.91 (0.45, 1.85)
**Cheaper cigarette brands**	51 (26.4)	123 (15.5)	32 (15.8)	1.27 (0.72, 2.24)	0.62 (0.29, 1.31)
**Online purchasing^a^**	4 (3.7)	20 (1.9)	5 (5.0)	0.86 (0.19, 3.87)	**4.12 (1.07, 15.82)**
**Purchasing frequency**	81 (46.9)	76 (11.1)	10 (6.1)	**5.19 (2.95, 9.11)**	0.40 (0.12, 1.33)
**From Indian Reservation^a^**	17 (10.2)	57 (5.8)	9 (3.9)	1.47 (0.66, 3.24)	0.56 (0.15, 2.13)
**Fewer Cigarettes than normal^a^**	11 (8.1)	86 (9.6)	132 (71.9)	0.56 (0.20, 1.54)	**29.35 (17.45, 49.36)**
**Other tobacco products^a^**	15 (10.6)	41 (6.2)	11 (7.6)	0.83 (0.30, 2.32)	0.49 (0.15, 1.58)

n = Unweighted Frequency; % = Weighted Row Percentages.

aOR: Adjusted Odds Ratio.

CI: 95% Confidence Interval.

¶ Adjusted for all other cigarette purchasing behaviors, nicotine addiction, smoking behavioral group, age, gender, race, household income, state of residence, and employment status.

a: One response missing in total sample size.

All cigarette purchasing behaviors reported as Yes vs No.

Bolded odds ratios are significant at the alpha less than or equal to 0.05.

### Qualitative study: Focus groups

#### Participant characteristics

Among the total focus group sample (n = 61), 61% of participants were female, 44% were aged 45–61 years, and 61% were White, Non-Hispanic. About 55% had at least a college degree, and 66% had an annual household income of at least $50K (Table 6).

**Table 6:** Participant characteristics (focus groups).

Participant Characteristic	Current Exclusive Smokers N = 16, n(%)	Transitioning Smokers/E-cigarette users N = 23, n(%)	Current E-cigarette users N = 22, n(%)	Total Sample N = 61, n(%)	P^†^ Value
**Age group**					
**21–29**	0	4 (17.4)	7 (31.8)	11 (18)	.182
**30–44**	9 (56.3)	7 (30.4)	7 (31.8)	23 (37.7)	
**45–61**	7 (43.8)	12 (52.2)	8 (36.4)	27 (44.3)	
**Sex**					
**Male**	1 (6.3)	13 (56.5)	10 (45.5)	24 (39.3)	**.005**
**Female**	15 (93.8)	10 (43.5)	12 (54.5)	37 (60.7)	
**Race**					
**NH, White**	11 (68.8)	13 (56.5)	13 (59.1)	37 (60.7)	.518
**NH, Black**	4 (25.0)	6 (26.1)	8 (36.4)	18 (29.5)	
**Other, HP**	1 (6.3)	4 (17.4)	1 (4.5)	6 (9.8)	
**Education**					
**Less than High School**	1 (6.3)	0	0	1 (1.6)	.546
**High School Graduate**	2 (12.5)	4 (17.4)	1 (4.5)	7 (11.5)	
**Some College or Technical School**	5 (31.3)	7 (30.4)	7 (31.8)	19 (31.1)	
**College Graduate**	4 (25.0)	9 (39.1)	11 (50)	24 (39.3)	
**Graduate degree**	4 (25.0)	3 (13)	3 (13.6)	10 (16.4)	
**Household Income**					
**Under $25K**	1 (6.3)	2 (8.7)	0	3 (4.9)	.094
**$25K-$34,999**	1 (6.3)	5 (21.7)	2 (9.1)	8 (13.1)	
**$35K-$49,999**	6 (37.5)	1 (4.3)	3 (13.6)	10 (16.4)	
**$50K-$74,999**	4 (25.0)	5 (21.7)	8 (36.4)	17 (27.9)	
**$75K-$99,999**	3 (18.8)	3 (13.0)	6 (27.3)	12 (19.7)	
**$100K-$149,999**	1 (6.3)	7 (30.4)	2 (9.1)	10 (16.4)	
**$150K+**	0	0	1 (4.5)	1 (1.6)	

NH- Non-Hispanic; HP- Hispanic; N = Frequency; % = Row percentages.

† Pearson Chi-Square test used to compute P values for Age group, Gender, Race, Education, and Household Income.

Bolded P values are statistically significant at 0.05.

#### E-cigarette and cigarette purchasing behavior

Many e-cigarette users in the focus groups reported to have not changed their e-cigarette purchasing behavior during the COVID-19 pandemic. For a few, changes in purchasing behavior included changes in purchasing location from retail vape shops to online, grocery stores, or gas stations and changes in purchasing quantity through buying more packs or cartons per store visit. Some of the factors perceived to be responsible for these changes were fear of exposure to COVID-19, reducing trips to multiple locations by purchasing everything from a single grocery store or looking for cheaper prices. Current e-cigarette users reported increasing their purchasing quantity only during the initial phase of the pandemic, which normalized later as they found alternative purchasing locations with better price deals such as online stores.

Exclusive smokers commonly reported looking for better deals on tobacco products, including coupons and places that sell cheaper tobacco products. Many of them reported purchasing the available tobacco products in bulk and a few others reported buying other tobacco product brands when their brand of choice was unavailable. For example, some smokers reported buying multiple shorter-sized cigarettes because longer ones were unavailable. Soaring prices during the early pandemic, limited availability of tobacco products, and limited store hours led to perceptions of reduced accessibility of tobacco products of choice for current smokers. This perception of limited accessibility was reported as a major factor responsible for the change in cigarette purchasing behaviors. In addition, a few exclusive smokers and transitioning smokers or e-cigarette users concerned about their increasing smoking during the pandemic, mentioned that they reduced their purchasing frequency and quantity during the COVID-19 pandemic. On the other side, those reporting bulk purchasing of tobacco products did not mention any concern regarding their smoking frequency during the pandemic (Table 7).

**Table 7:** Themes identified in the qualitative analysis (focus group study).

Theme	User Group	Quote
**No change in purchasing behavior**	Current E-cigarette users	Male, 48, White: *I haven’t noticed any problems when I go to buy or purchase any of it*. *I normally buy from the convenience store*. *And I haven’t had a reason for them to change on price nor quantity*
		Female, 55, Black: *“About the same*. *Yeah*. *It’s about the same for me as well*. *I mostly go to Sam’s*. *There’s always a long line*, *but yeah*, *it’s about the same*.*”*
**Change in purchasing location**	Current E-cigarette users	Female, 26, White: *I mean*, *I guess you could say that if I found myself at the gas station*, *either if I was getting gas or if I was at the grocery store or something*, *I would try to kill two birds with one stone and get what I need when I’m there and maybe even extra because of that*
**Change in purchasing behavior during the initial pandemic only**	Current E-cigarette users	*Male*, *23*, *Asian*: *“I think for me*, *it was only during March when it first started beginning*. *Everyone thought everything was going to close down*, *they thought the world was going to end*, *so they would buy out the whole shop*. *So*, *once I had found out things are cheaper online*, *I think I started leaning more towards that instead of going to the actual stores and buying stuff*.*”*
**Bulk purchasing**	Transitioning Smokers / E-cigarette users	Male, 21, Asian: *“Ever since [the pandemic] … I bulked up on my quantity [of E-CIGARETTE products]*, *so that way I run out less*.*”*. *“I’ll buy two*, *four packs*, *just the last weeks*, *so I don’t have to go out for a while”*.
	Exclusive Smokers	Female, 43, More than one race: *“I’ll buy it in a greater amount*, *and so I don’t have to go back out”*
	Exclusive Smokers	Female, 54, White: *“For me*, *regarding smoking*, *I was so nervous about being able to get out*. *I went from buying a pack a day to two cartons for the next two weeks because I didn’t want to be out on my supplies*.*”*
**Reduced purchasing frequency**	Exclusive Smokers	Female, 40, White: *“I don’t buy a carton just because I feel like if I was to buy a carton*, *I would smoke more and I don’t need to smoke more than I am*.*”*
	Transitioning Smoker / E-cigarette user	Male, 47, Black: “*Since COVID*, *it’s like you don’t know what to do when you get to certain places*. *They’re not really wearing masks*, *and there’s no social distancing*, *and you’re like*, *’Well can’t make a purchase here now*,*’ so you just get back in your car and go home*.*”*
**Changing tobacco product brands**	Exclusive Smokers	Female, 60, White: *“Well*, *the cigarettes I smoke are Capris*, *and they’re really long*. *The last time I went to go get it*, *they didn’t have the long ones*, *so I had to get the short ones*. *I noticed*, *even though they’re supposedly the same thing*, *they were a lot stronger*.*”*
	Transitioning Smoker / E-cigarette user	Female, 29, Other: *“I didn’t think much about it*. *But I know the gas stations and stuff right by my house*, *they started getting a smaller and smaller supply*. *A lot of things were sold out and then happened to choose alternatives*. *I think I went and they didn’t have any of the Blu cartridges so I had to buy one of these random vapes if I wanted*.*”*
**Searching for cheaper products and places that sell at lower price**	Exclusive Smokers	Female, 57, White: *“Yes*. *I have to look for the cheaper places now because I’m buying more*.*”*
	Exclusive Smokers	Female, 40, White: *“But I just look for specials sometimes I just go online*, *look for coupons”*.
	Current E-cigarette users	Male, 23, Asian: *“Ever since everything closed down*, *you had to look online for your products and stuff now*. *So*, *once I had found out things are cheaper online*, *I think I started leaning more towards that instead of going to the actual stores and buying stuff*.*”*
**Purchasing behavior affecting the use of tobacco products**	Exclusive Smokers	Female, 44, White: *“I was doing it by the carton*, *but we were smoking more*. *So now I have us buy a three-pack special every couple of days and it kind of keeps me in check and him in check*, *like here’s my pack*, *here’s your pack*, *we’re going to share this third pack”*.
	Transitioning Smoker / E-cigarette user	Male, 47, White: *“I found myself buying more at each visit because of what I spoke about earlier and just for the convenience of not having to go back to the store as often*.*”*

## Discussion

We examined the changes in tobacco purchasing behaviors that occurred during the COVID-19 pandemic among US adults using a mixed methods approach. Our survey findings show that many participants reported no change in their purchasing behavior in the context of the pandemic. But among those who did change, dual users as compared to exclusive smokers were more likely to look for other tobacco products, buy fewer cigarettes than normal, buy cheaper cigarette brands, and buy from Indian Reservations. We also found that the odds of increased e-cigarette use were higher for those who increased their purchasing frequency and for those who changed their e-cigarette product brand, while the odds of increased smoking were higher for those who bought more cigarette packs per store visits and went to stores more frequently to buy cigarettes, after controlling for nicotine addiction, tobacco user groups, residence state, and socio-demographic variables. In addition, the odds of decreased smoking were higher for those current smokers who bought fewer cigarettes than normal. These findings were informed by the results of the focus group discussions, offering valuable insights on factors such as perceptions of reduced accessibility due to store closures and lockdown, fear of exposure to COVID-19 infection, and price changes during the pandemic driving different tobacco purchasing behaviors among tobacco users.

Most findings from the focus group study were consistent with the quantitative survey findings and provided valuable insights on the potentially associated factors driving the change in the evident purchasing behaviors. Survey findings showed that smoking frequency was associated with the amount for cigarettes purchased. These findings were validated by exclusive smokers from focus groups who mentioned that they observed increase in their smoking when they bulk purchased cigarettes. Moreover, transitioning smokers or e-cigarette users who are attempting to quit reported to have controlled their purchasing frequency consciously by buying fewer cigarettes during the pandemic. Bulk purchasing was frequently mentioned in the focus groups. These findings are consistent with the previously published literature that reports increased cigarette sales during pandemic [16,29].

In addition to bulk purchasing, studies conducted during the pandemic found online purchasing as a significant tobacco purchasing behavior during the pandemic [14]. Consistently, online purchasing was also frequently reported by participants in our focus group study. Findings from our survey suggests that dual users are more likely to purchase tobacco products online as compared to exclusive smokers. On the other hand, focus group discussion highlights factors such as cheaper prices online or convenience to shop from home as major factors driving the online purchasing behavior during the pandemic.

From our survey, dual users as compared to exclusive users had higher odds of buying alternate tobacco products. Consistently, findings from focus groups also suggest that dual users such as current e-cigarette users (who may also be smoking cigarettes and not trying to quit smoking) or transitioning smokers or e-cigarette users were more open to buying other tobacco products due to the unavailability of their preferred product, while exclusive smokers seemed to be making extra efforts to buy their product of choice rather than switching to alternate tobacco products that are available. Since dual users have experience using both products (e-cigarettes and cigarettes), they can switch between the products at any time in an attempt to satisfy their nicotine needs. Having the choice to get nicotine from somewhere else, such as from an alternate tobacco product or place of purchase, when their usual product is either expensive or non-available during the pandemic, can lead to a change in their purchasing behavior which is evident from our findings. These findings are consistent with previously conducted studies [16] that reports increased alternative tobacco product use during the COVID-19 pandemic.

Studies have found that dual use can be considered as a transitional state leading to switching completely to e-cigarettes [30] and factors such as health concerns and stress can influence tobacco use during pandemic situations [12]. Thus, dual users buying fewer cigarettes than normal during the COVID-19 pandemic could be attributed to their motivations to quit smoking during the pandemic. On the other hand, a study found that exclusive users are more likely to perceive harm from other tobacco products than dual users [31] and this could be one of the reasons why exclusive users are less likely to change their purchasing behavior and like to stick to their usual product for nicotine needs instead of exploring alternate tobacco products.

Findings from the focus group also suggest that these purchasing behaviors differed over time. Purchasing was reported to be more frequent and included large quantities at the start of the pandemic, possibly because of the perceived scarcity of tobacco products, but eventually reduced or normalized either due to restocking of the products in the stores or concerns over their increasing tobacco use due to large quantity bought. These qualitative findings hint at a bidirectional relationship between tobacco purchasing and tobacco use; these mutual influences that change over time need to be further explored in longitudinal studies.

## Limitations

First, the quantitative survey data were self-reported by the participants who were asked to retrospectively answer the questions, which may have resulted in recall bias. However, the study was conducted during the pandemic period when participants could still have fresh memories of their experiences during the pandemic, thus minimizing recall bias [1]. Second, the survey was a cross-sectional study and thus we could not determine causal relationships. Third, the qualitative study, which used a different sample than the quantitative study, was not representative of the US population. However, the use of two different samples for quantitative survey and focus groups is a strength because it allowed us to capture a wider range of experiences and perspectives, leading to richer data and uncovering themes not visible within a single sample. It improves the methodological rigor by allowing for the triangulation of results across different groups, strengthening the validity and reliability of our conclusions and reducing the risk of bias. Fourth, some of the questions from the quantitative survey about purchasing behaviors of cigarettes and e-cigarettes were newly developed by the research team for this study and were not tested thoroughly for accurate validation. Fifth, there could be differences in the tobacco control environment based on locally implemented tobacco laws. Due to the small sample size, we could not control for these local tobacco control laws in the analysis, however, we do control for residence state to minimize these differences. Further studies would be needed in order to determine how large of an effect local and Tobacco-21 tobacco laws may have had on behavior. And finally, it is possible that some non-significant associations in the quantitative survey were due to an insufficient number of observations in the analysis, reflected by a large confidence interval of the estimated OR. However, small cell sizes would not affect significant associations found in our study and our conclusions. The sample size for focus groups was small as the participants were required to be able to attend sessions remotely. This may have introduced selection bias as those without the technical capabilities were eliminated.

## Conclusion

During stressful situations that may affect the availability of the preferred tobacco products such as the COVID-19 pandemic, dual users of both cigarettes and e-cigarettes are more likely to change their tobacco purchasing behaviors as compared to exclusive users of tobacco products. To be able to switch between tobacco products when in need of nicotine, perceptions of reduced accessibility, changes in prices, fear of getting infected, and convenience may have driven some of the purchasing behavior changes seen in dual users as opposed to exclusive users and this should be monitored during stressful societal situations. These difference in purchasing behaviors between tobacco user groups such as dual users as compared to exclusive users can help in the development of targeted tobacco control strategies and policies for different tobacco user groups.

## Implications

The actual and perceived disruptions to the supply of tobacco products during the COVID-19 pandemic are associated with the purchasing behaviors of different tobacco user groups and can influence their tobacco use. Educational campaigns can target different tobacco user groups separately and can promote choosing reduced-risk tobacco products among dual users. Studies have found that cigarette-only users reported higher intention to quit smoking than e-cigarette-only users during the pandemic [32]. Stressful socially disruptive situations might be an opportune time for doctors and public health workers to educate smokers and dual users about the benefits of quitting smoking (including both health-related and financial), controlling their purchasing behaviors, and directing them toward smoking cessation resources. The findings from this study could help policymakers to develop policies monitoring and controlling the sale of tobacco products considering the purchasing behaviors of the tobacco product users during the stressful situations such as pandemic, for example, limiting the amount of tobacco products purchased at a time per person.
